# Effects of nano-selenium and/or vitamin E supplementation on growth performance, antioxidant status, histopathology and resistance to *Aspergillus flavus* in Nile tilapia (*Oreochromis niloticus*)

**DOI:** 10.1186/s12917-025-04471-y

**Published:** 2025-02-05

**Authors:** Walaa K. Bazina, Wesam A. Tawfik, Nadia A. Abd Elghany, Saadea Saadony, Zulhisyam Abdul Kari, Muna Omer Alamoudi, Mohamed Y. M. Aly, Abuelhassan Elshazly Younis, Roshmon Thomas Mathew, Moaheda E. H. Eissa, Mohammad Bodrul Munir, Saadiah Ibrahim, Ismail Yousef, Yusuf O. H. Omar, El-Sayed Hemdan Eissa, Heba E. Abd Elnabi

**Affiliations:** 1 National Institute of Oceanography and Fisheries (NIOF), Cairo, Egypt; 2 NaQaa Nanotechnology Network (NNN), Giza, Egypt; 3 Fish Diseases Department, Animal Health Research Institute, Dokki, ARC, Giza Egypt; 4 Department of Animal Production and Fish Resources, Faculty of Agriculture, Suez Canal University, Ismailia, Egypt; 5 Department of Agricultural Sciences, Faculty of Agro-Based Industry, Universiti Malaysia Kelantan, Jeli Campus, Jeli, 17600 Malaysia; 6 Biology Department, Faculty of Science, University of Ha’il, Ha’il, Saudi Arabia; 7 Biology Department, Faculty of Science at Yanbu, Taibah University, Yanbu El-Bahr, 46423 Kingdom of Saudi Arabia; 8 Fish Resources Research Center, King Faisal University, Hofuf-420, Al-Ahsa, 31982 Saudi Arabia; 9 Biotechnology Department, Fish Farming and Technology Institute, Suez Canal University, Ismailia 41522, Egypt; 10 Faculty of Agriculture, Universiti Islam Sultan Sharif Ali, Sinaut Campus, Tutong, TB1741 Brunei Darussalam; 11 Fisheries Research Institute Pulau Sayak, Department of Fisheries Malaysia, Ministry of Agriculture and Food Security, 08500 Kota Kuala Muda, Kedah, Malaysia; 12 Aquaculture Consultant, Mansoura, Egypt; 13 Department Medicine and Surgery, Faculty of Medicine, Mogadishu University, Mogadishu, Somalia; 14 Fish Research Centre, Faculty of Environmental Agricultural Sciences, Arish University, El-Arish, Egypt; 15 Department of Fish Resources and Aquaculture, Faculty of Environmental Agricultural Sciences, Arish University, El-Arish, Egypt

**Keywords:** Selenium nanoparticles, Vitamin E, *Oreochromis niloticus*, Body composition, Biochemical parameters, Histopathology, *Aspergillus flavus*

## Abstract

**Background:**

This study aimed to evaluate the effects of selenium nanoparticles (SeNPs) and/or vitamin E (VE) on the growth, body composition, metabolic parameters, histopathology, and resistance of Nile tilapia to *Aspergillus flavus*.

**Results:**

Monosex Nile tilapia fingerlings were sourced from the Bazina farm and hatchery in Ismailia Governorate, Egypt, where the experiment was also conducted. The fish were acclimatized for 15 days before the trial. A total of 240 fingerlings (average weight 46 ± 3.0 g/fish) were divided equally across 12 concrete tanks (1 × 1 × 1.2 m, 1 m³ capacity), with 20 fish per tank. The fish were fed a control diet (T0), which was a basal diet with no supplementation, or one of three experimental diets for 60 days: T1 (1 mg SeNPs/kg), T2 (100 mg VE/kg), and T3 (1 mg SeNPs + 100 mg VE/kg). The experiment followed a completely randomized design (CRD) with three replicates per treatment. The combination of SeNPs and VE (T3) resulted in the best feed conversion ratio. A slight but significant increase (*P* *< 0.05*) in whole-body composition was observed in all treatment groups compared to the control. Biochemical parameters, serum digestive enzyme activity, and antioxidant levels improved significantly with dietary supplementation. Histopathological analysis revealed somewhat lacerated gill arches in fish fed SeNPs, VE, or their combination, but the overall gill structure remained normal. The SeNPs + VE group exhibited improved villi length and normal morphology of portal veins and hepatic sinusoids, though some vacuolated hepatocytes were noted. Fish in the SeNPs + VE group had the lowest mortality rates and the highest resistance to *A. flavus*.

**Conclusion:**

Supplementing diets with SeNPs and/or VE enhances growth, body composition, biochemical parameters, and histopathology in Nile tilapia. The combination of 1 mg SeNPs + 100 mg VE/kg improves immune response and growth, offering a promising strategy to enhance Tilapia health and productivity.

## Background

Animal protein for human consumption comes primarily from the fisheries and aquaculture industries [1]. As more countries depend on fish as a key food source, fish farming is vital for meeting global food demands [2, 3]. In 2018, global aquaculture production reached 82.1 million tons, making up about 46% of total fish production [4]. Nile tilapia (*Oreochromis niloticus*) is one of the most widely farmed freshwater fish globally. Its competitive market price, fast growth, ease of breeding, high productivity, fecundity, stress tolerance, and ability to thrive in various environmental conditions make it an essential species [5–8]. Nile tilapia is highly popular in Egypt and worldwide [9, 10]. Tilapia production is one of the fastest-growing sectors in global aquaculture [11]. A well-balanced diet for aquatic animals includes minerals, vitamins, proteins, lipids, and carbohydrates, with micronutrients like selenium playing a key role in fish health [12, 13]. Selenium, even in small amounts, is essential for various physiological processes, protecting cell membranes from oxidative damage and supporting growth, development, and antioxidant defense through selenoprotein production [14–16].

Nanotechnology is increasingly applied in aquaculture, particularly through the use of nanotrace elements like selenium in aquafeeds [17]. Nanoparticles, with their larger surface area and faster interaction with organic molecules, enhance gastrointestinal absorption in fish [18–24]. Selenium NPs offer advantages such as improved chemical stability, lower toxicity, and gradual selenium release, which boosts bioavailability and production efficiency [6, 25]. Studies have shown that adding SeNPs to aquafeeds improves fish growth, feed efficiency, and antioxidant capacity [17].

Vitamin E is a crucial lipid-soluble antioxidant that protects cell membrane lipids from oxidation by neutralizing free radicals, particularly lipid peroxyl radicals [26, 27]. It supports fish health by enhancing immune response, stress tolerance, and disease resistance [28, 29]. Since fish cannot synthesize (VE), dietary supplementation is vital to prevent deficiencies that can cause lipid oxidation, anemia, liver and muscle degeneration, and even death [30, 31]. Moreover, vitamin E works synergistically with selenium to protect cell membranes from lipid peroxidation [16, 32].

Fish diseases cause economic losses [33, 34]. Fungal infections reduced hatchability, slower growth, and mycotoxin contamination in feed [35, 36]. *Aspergillus* spp., classified as secondary pathogens, lead to systemic diseases with high mortality rates, primarily spread through contaminated feed [37, 38]. Studies highlight the synergistic effects of VE and selenium as crucial micronutrients in fish, influencing immunity [26], growth [39], and protection against oxidative stress [40]. Few studies have examined the effects of SeNPs or VE alone or in combination, on growth, immunity, blood health, and disease resistance in fish. This study aimed to assess the feasibility of supplementing Nile tilapia diets with nano-selenium or vitamin E and to evaluate their impact on growth, biochemical parameters, histology, and resistance to *Aspergillus flavus*.

## Methods

### Synthesis and characterization of (SeNPs)

Selenium (5 mm, > 99.99%) was supplied by Sigma-Aldrich. The (SeNPs) were synthesized using the high-energy ball milling technique, as described by Naiel et al. [41]. The particle size, calculated using Scherrer’s equation, was approximately 24 nm.

### Experimental design and feed protocols

The present study was conducted in a private aquaculture farm under natural conditions with prior approval of the Arish University Research Ethics Committee (Code ARU/Agri, 23). A total of 12 concrete tanks (1 × 1 × 1.2 m, capacity 1 m^3^). The average body weight of monosex Nile tilapia fish is 46 ± 3.0 g/fish. A basal diet with 0 mg of SeNPs/kg of food or VE supplementation was utilized as a control (T0) = 0.0 mg (SeNPs /VE)/kg. T1 = 1 mg SeNPs /kg, T2 = 100 mg VE/kg, and T3 = 1 mg SeNPs + 100 mg VE/kg are the three used different diets according to [42].

The selenium nanoparticles and vitamin E (Vit E, 400 mg, Pharco Pharmaceuticals, Egypt) was provided by the Egyptian pharmaceutical company) were dispersed in 100 mL of distilled water, uniformly sprayed onto the dietary components, thoroughly mixed for 30 min, and subsequently pelleted to a diameter of 2 mm. The prepared diets were stored in plastic bags at − 4 °C for further use. Two hundred forty monosex Nile tilapia were obtained from the Bazina farm and hatchery in Ismailia Governorate, Egypt, and acclimated in the same facility for 15 days. After the acclimation period, the 240 healthy Tilapia were randomly assigned to 12 concrete tanks. In these tanks, the fish were divided into four groups, with each group consisting of three replicates (20 fish per tank).

For sixty days, the fish were manually fed the experimental diets three times daily at 07:00, 11:00, and 15:00 h. The feeding rate was set at 3% of the fish’s body weight, with the amount of feed adjusted based on their weight. Each tank received continuous aeration through blowers, with an aeration hose connected to an aeration stone. Prior to the daily water change, the aeration was turned off, and waste was removed from the tank bottom. The water change involved replacing 25% of the water with fresh water once a day before the first meal. The experimental diets were formulated to meet the nutritional requirements of tilapia, as outlined by the National Research Council [43]. The nutritional components and approximate composition of the diets are presented in Table 1. The mean water temperature, pH, dissolved oxygen, ammonia, nitrite, and nitrate levels were 28.6 ± 0.41 °C, 6.97 ± 0.33, 5.52 ± 0.21 mg L⁻¹, 0.23 mg L⁻¹, 0.0071 ± 0.002 mg L⁻¹, and 9.39 ± 0.012 mg L⁻¹, respectively. These water quality parameters were consistently maintained throughout the study.

**Table 1:** Formulation of four experimental diets (g/kg) for Nile Tilapia

Ingredient	Experimental diets			
	Control	SeNPs	Vit E	SeNPs + Vit E
Fish meal (CP 72%)	110.0	110.0	110.0	110.0
Rice bran	200.0	200.0	200.0	200.0
Soybean meal (CP 48%)	360.0	360.0	360.0	360.0
Wheat bran	200.0	200.0	200.0	200.0
Yellow corn	60.0	59.999	59.990	59.989
SeNPs	-	0.001	-	0.001
Se	-	-	-	-
Vit E			0.010	0.010
Fish Oil (herring)	15.0	15.0	15.0	15.0
Soybean Oil	15.0	15.0	15.0	15.0
Molasses	20.0	20.0	20.0	20.0
Dicalcium phosphate	10.0	10.0	10.0	10.0
Premix	10.0	10.0	10.0	10.0
Total	1000	1000	1000	1000
Se content (calculated) mg/kg diet	1.134	1.134	1.134	1.134
**Calculated composition**				
Dry matter	91.41	91.42	91.46	91.43
Crude protein	31.4	31.4	31.41	31.4
Crude fat	8.17	8.16	8.17	8.17
Ash	7.23	7.24	7.21	7.23
NFE	38.04	37.98	38.10	38.08

Se free premix: each 1 kg had 8600 IU; Vitamin mixture (except (VE), mg kg^− 1^ premix) vitamin D3, 580,000 IU vitamin A, 142 mg vitamin K3, 0.1 mg vitamin C, 720 mg vitamin B2, 58 mg vitamin B1, 58 mg vitamin B12, 86 mg folic acid, 34 mg vitamin B6, 8 mg pantothenic acid, 2000 mg iron sulfate, 3000 mg zinc methionine, 65 mg manganese sulfate, 25 mg calcium iodide, 3400 mg copper sulfate, 572 mg cobalt. Vit E; (VE), Se; selenium, SeNPs; selenium nanoparticles, CP; crude protein, DE; nutritional digestibility, NFE; nitrogen-free extract. Trouw Nutrition Company, El Salam Road, Belbies, Sharkia, Egypt, supplied the feed ingredients

### Growth performance and feed utilization parameters

At the end of the experiment, fish from each concrete tank were weighed to assess growth performance characteristics, following the procedure outlined by Eissa et al. [38].

Weight gain (WG; g/fish) was calculated as the difference between final body weight and initial body weight.

Average daily gain (ADG) (g/fish /day) = (Final body weight (FBW)- Initial body weight (IBW))/ duration (day).

Specific growth rate (%/day): SGR = 100 × [(ln FBW - ln IBW) / days].

Survival (%) = 100 × (final number of fish / initial number of fish).

Feed conversion ratio (FCR) = Feed intake (g) / Weight gain (g).

Feed Intake (g/fish): The amounts of feed consumed throughout the investigational period/fish (g) or Feed Intake (g/fish) = (Initial weight × daily feed rate) × the feeding days number.

### Chemical analyses

Five fish were randomly selected from each treatment for a comprehensive analysis of the whole fish body. The crude protein, crude lipid, and ash contents were determined following AOAC [44] methods. To measure moisture content, the samples were dried in a drying oven (GCA, model 18 EM, Precision Scientific Group, Chicago, IL, USA) at 85 °C for 24 h.

### Blood sampling

At the end of the feeding trial, all fish were anesthetized using 150 mg/L MS222 [45]. Blood samples were collected from the caudal blood vessels of three fish per tank using a syringe-free of anticoagulants. The clotted blood was then centrifuged at 3000 rpm for 15 min at 4 °C to obtain serum, which was stored at -20 °C.

### Biochemical parameters

Using premade chemicals (Spinreact Co. Spain kits) and the manufacturer’s instructions, Henry’s methods for determining the serum levels of total protein (ID. No. MBS9917835) and albumin (ID. SB-028-500), Trinder’s methods assessed glucose levels (ID. No. 1001192) [46], alanine aminotransferase (ALT, ID. MBS038444), aspartate aminotransferase (AST, Catalog No. EK12276), alkaline phosphatase (ALP, ID. MBS033204), and creatinine (Human Co., Germany, ID. No. 10051) were measured using the RA-50 chemistry analyzer (Bayer). Triglyceride and cholesterol levels (Spinreact Co., Santa Coloma, Spain, ID. No. 1001091) were determined using a spectrophotometer (Lambda EZ201; Perkin Elmer). Examination of digestive enzyme activities in serum involved determining the presence of the enzymes lipase, protease, and amylase using the method described by Hidalgo et al. [47].

### Immune response

Lysozyme activity was measured in µg mL^− 1^ using the Parry et al. (1965) method. The following technique was used to evaluate the phagocytic activity: Phagocytosis (%) = $\:\frac{\text{T}\text{h}\text{e}\:\text{n}\text{u}\text{m}\text{b}\text{e}\text{r}\:\text{o}\text{f}\:\text{p}\text{h}\text{a}\text{g}\text{o}\text{c}\text{y}\text{t}\text{i}\text{c}\:\text{h}\text{e}\text{m}\text{o}\text{c}\text{y}\text{t}\text{e}\text{s}}{\text{T}\text{o}\text{t}\text{a}\text{l}\:\text{n}\text{u}\text{m}\text{b}\text{e}\text{r}\:\text{o}\text{f}\:\text{h}\text{e}\text{m}\text{o}\text{c}\text{y}\text{t}\text{e}\text{s}\:}\:\times\:\:100$ [42].

### Antioxidant analyses

The activities of superoxide dismutase (SOD) and catalase [48] in fish serum were measured using diagnostic reagent kits, following the manufacturer’s instructions (Cusabio Biotech Co., Ltd; China). Malondialdehyde (MDA) level was measured using the same diagnostic reagent kits [42].

### Challenge test

A strain of *Aspergillus flavus* was obtained from the Microbiological Unit of the Fish Diseases Department at the Animal Health Institute in ARC Dokki, Giza. All fish (two replicates per group, with 10 fish per replicate) were challenged with *A. flavus* after the final sampling. The fish were injected intraperitoneally with *A. flavus* (4 × 10³ conidia/ml) and observed for 15 days in aquariums (125 L) [38]. To confirm the re-isolation and identification of the fungus, specimens from the liver, gills, and muscles of both live and deceased fish were analyzed under a microscope and inoculated onto potato dextrose agar (PDA) plates at the end of the experimental challenge [49]. As previously mentioned, the cumulative mortality rate (%) was calculated [50].

### Histopathology assays

After the challenge test, three fish from each aquarium were selected, and their gills, liver, and intestines were collected at the end of the experiment. These tissues were preserved in Bouin’s solution for approximately twenty-four hours. The fish had been maintained in an isotonic saline solution. After standard preservation in 70% ethyl alcohol, the specimens were dehydrated through a series of alcohol grades, cleared in xylene, and embedded in paraffin wax. Thin Sect. (6 μm) were placed on chemically pure glass slides. Following Pearse [51], the sections were prepared and stained with Harris’s Hematoxylin and Eosin. Biometric indices, including villus height (µm), villus width (µm), muscular layer thickness (µm), and goblet cell count, were measured using micrometer slides on each segment of the intestine.

### Statistical analysis

The means and standard errors of the data are presented. Statistical analysis was performed using SPSS version 26. One-way ANOVA was applied to evaluate the data, and mean differences were assessed using the Duncan test at the 0.05 probability level [52].

## Results

### Growth performance assays

In the feeding trial conducted over 60 days, the SeNPs + (VE) (SeNPs + VE) group outperformed the other groups, showing greater final body weight, weight gain, average daily gain (ADG), specific growth rate (SGR), and fish biomass. The VE group consumed more feed than the other groups. The SeNPs + VE group achieved the best FCR, followed by SeNPs, VE, and the control group in that order. Survival rates remained consistent across all groups (Table 2).

**Table 2:** Growth (Mean ± SE) performance and survival rate of tilapia after 60 days feeding trial

Parameter	Control	SeNPs	VE	SeNPs + VE
Initial weight (g)	46.13 ± 0.12^a^	46.17 ± 0.07^a^	46.43 ± 0.19^a^	46.13 ± 0.09^a^
Final weight (g)	90.90 ± 1.25^d^	98.83 ± 0.73^c^	102.87 ± 1.02^b^	107.13 ± 1.19^a^
Weight gain	44.77 ± 1.13^d^	52.67 ± 0.67^c^	56.43 ± 1.18^b^	61.00 ± 1.10^a^
Feed intake	71.97 ± 0.19^a^	72.02 ± 0.10^a^	72.44 ± 0.29^a^	71.97 ± 0.14^a^
FCR	1.61 ± 0.04^a^	1.37 ± 0.02^b^	1.28 ± 0.03^b^	1.18 ± 0.02^c^
SGR	1.13 ± 0.02^d^	1.27 ± 0.01^c^	1.33 ± 0.02^b^	1.40 ± 0.02^a^
ADG	0.75 ± 0.02^d^	0.88 ± 0.01^c^	0.94 ± 0.02^b^	1.02 ± 0.02^a^
Survival rate	100.00 ± 0.0	100.00 ± 0.00	100.00 ± 0.00	100.00 ± 0.00

Superscript indicates the significance (*P* *< 0.05*) differences among the experimental diets

### Chemical composition

Table 3 shows a slight increase (*P* *< 0.05*) in the whole-body composition of Nile tilapia across all treatment groups compared to the control group. This includes moisture, crude protein, crude lipid, and ash content.

**Table 3:** Chemical composition (%) of whole-body tilapia in four formulated feeds

Parameter	Control	SeNPs	VE	SeNPs + VE
Se Content	0.36 ± 0.01^b^	0.81 ± 0.00^a^	0.36 ± 0.00^b^	0.80 ± 0.00^a^
Moisture	77.06 ± 0.43^a^	77.23 ± 0.26^a^	77.63 ± 0.26^a^	77.11 ± 0.24^a^
Crude Protein	66.05 ± 0.36^a^	66.23 ± 0.23^a^	66.92 ± 0.28^a^	66.77 ± 0.09^a^
Crude Lipid	19.79 ± 0.06^c^	20.19 ± 0.07^b^	20.50 ± 0.13^ab^	20.66 ± 0.18^a^
Ash	13.63 ± 0.10^c^	14.35 ± 0.07^b^	14.64 ± 0.04^a^	14.75 ± 0.03^a^

Superscript indicates the significance (*P* *< 0.05*) differences among the experimental diets

### Biochemical parameters and immune response

The activities of ALT, AST, and ALP were lower in fish fed diets supplemented with SeNPs, VE, or a combination of both compared to the control group. The treatment groups showed higher levels of serum total protein, globulin, and albumin (except the VE group) compared to the control group. Table 4 indicates that the treatment groups had lower levels of creatinine, glucose, triglycerides, and cholesterol than the control group. Additionally, the treatment groups exhibited higher activities of lipase, protease, and amylase. Phagocytic and lysozyme activities were significantly higher in the treatment groups than in the control group (*P* *< 0.05*).

**Table 4:** Effect of four experimental diets on haemat-biochemical parameters and digestive enzymes activities of tilapia fish

Parameter	Control	SeNPs	VE	SeNPs + VE
ALT	8.24 ± 0.37^a^	6.94 ± 0.29^b^	4.94 ± 0.16^c^	6.80 ± 0.36^b^
AST	18.24 ± 0.21^a^	17.85 ± 0.29^a^	12.75 ± 0.23^b^	12.01 ± 0.21^b^
ALP	35.75 ± 0.62^a^	30.45 ± 0.55^bc^	31.47 ± 0.42^b^	29.68 ± 0.48^c^
TP	5.13 ± 0.20^b^	6.13 ± 0.12^a^	5.48 ± 0.03^b^	5.51 ± 0.06^b^
Albumin	2.88 ± 0.09^b^	3.24 ± 0.03^a^	2.67 ± 0.02^c^	3.04 ± 0.08^ab^
Globulin	2.25 ± 0.16^b^	2.90 ± 0.09^a^	2.80 ± 0.01^a^	2.46 ± 0.04^b^
Creatinine	0.54 ± 0.07^a^	0.47 ± 0.04^a^	0.49 ± 0.06^a^	0.43 ± 0.01^a^
Glucose	14.42 ± 0.59^a^	11.67 ± 0.36^b^	12.33 ± 0.20^b^	12.03 ± 0.33^b^
Protease	20.26 ± 1.11^c^	26.57 ± 0.65^b^	26.78 ± 0.33^b^	29.99 ± 0.70^a^
Lipase	32.28 ± 0.92^b^	34.08 ± 0.63^ab^	34.23 ± 0.85^ab^	35.28 ± 0.36^a^
Amylase	14.62 ± 0.17^b^	16.43 ± 0.50^a^	16.04 ± 0.08^a^	16.70 ± 0.27^a^
Cholesterol	189.08 ± 2.76^a^	167.14 ± 3.91^b^	166.27 ± 5.77^b^	153.16 ± 6.73^b^
Triglyceride	159.09 ± 4.40^a^	149.76 ± 3.89^ab^	136.29 ± 2.68^c^	142.34 ± 1.27^bc^
Lysozyme activity (u/ml)	27.12 ± 0.30^c^	29.61 ± 0.38^b^	30.05 ± 0.20^b^	31.41 ± 0.51^a^
Phagocytic activity	31.92 ± 0.41^c^	33.82 ± 0.48^ab^	32.60 ± 0.35^bc^	35.06 ± 0.28^a^

Superscript indicates the significance (*P* *< 0.05*) differences among the experimental diets

### Antioxidant activities

MDA levels decreased in the VE and SeNPs + VE groups but increased in the SeNPs group. SOD and CAT activities were higher in the treatment groups compared to the control group (Table 5).

**Table 5:** Effect of four experimental feeds on antioxidant activities of studied fish

Parameter	Control	SeNPs	VE	SeNPs + VE
SOD (IU/L)	15.18 ± 0.18^d^	18.03 ± 0.18^b^	16.79 ± 0.32^c^	18.95 ± 0.21^a^
CAT (IU/L)	11.68 ± 0.42^c^	14.18 ± 0.23^a^	12.81 ± 0.18^b^	14.05 ± 0.24^a^
MDA (IU/L)	26.59 ± 0.47^b^	28.45 ± 0.38^a^	24.50 ± 0.17^c^	25.18 ± 0.40^c^

Superscript indicates the significance (*P* *< 0.05*) differences among the experimental diets

### Histological assays

The present study conducted histological analyses on three tissues: gills, intestine, and hepatopancreas.

#### Gills

**Fig. 1:**
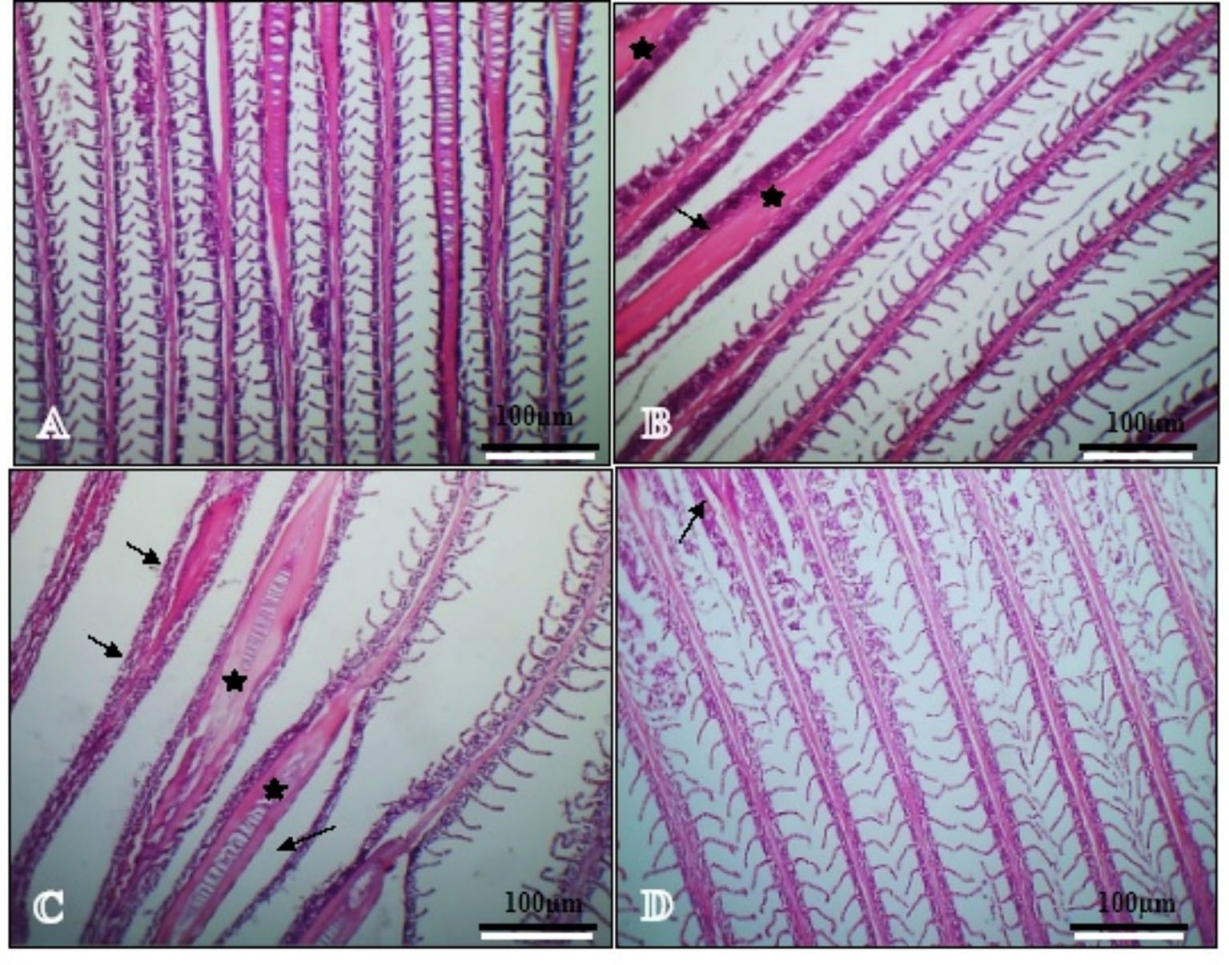
Transverse sections of fish gill show normal gill architecture control group (**A**), normal gill architecture with some laceration of gill arches (arrows) and hyperplasia and fusion of primary and secondary gill lamellae (stars) (Nano Se group (**B**); vitamin E group (**C**), normal gill architecture with light laceration of gill arches Nano Se + VE group (**D**) (H&E staining, at ×100 magnification, scale bar 100 μm)

No histological abnormalities were observed in the gills of Nile tilapia fed varying amounts of SeNPs and VE (Fig. 1). Fish fed the control diet displayed normal tissue architecture (Fig. 1A). Fish fed SeNPs, VE, and SeNPs + VE diets (Fig. 1B, C, and D) showed minor laceration of the gill arches, but otherwise, the gill architecture was normal, with hyperplasia and fusion of the primary and secondary gill lamellae.

#### Intestine

**Fig. 2:**
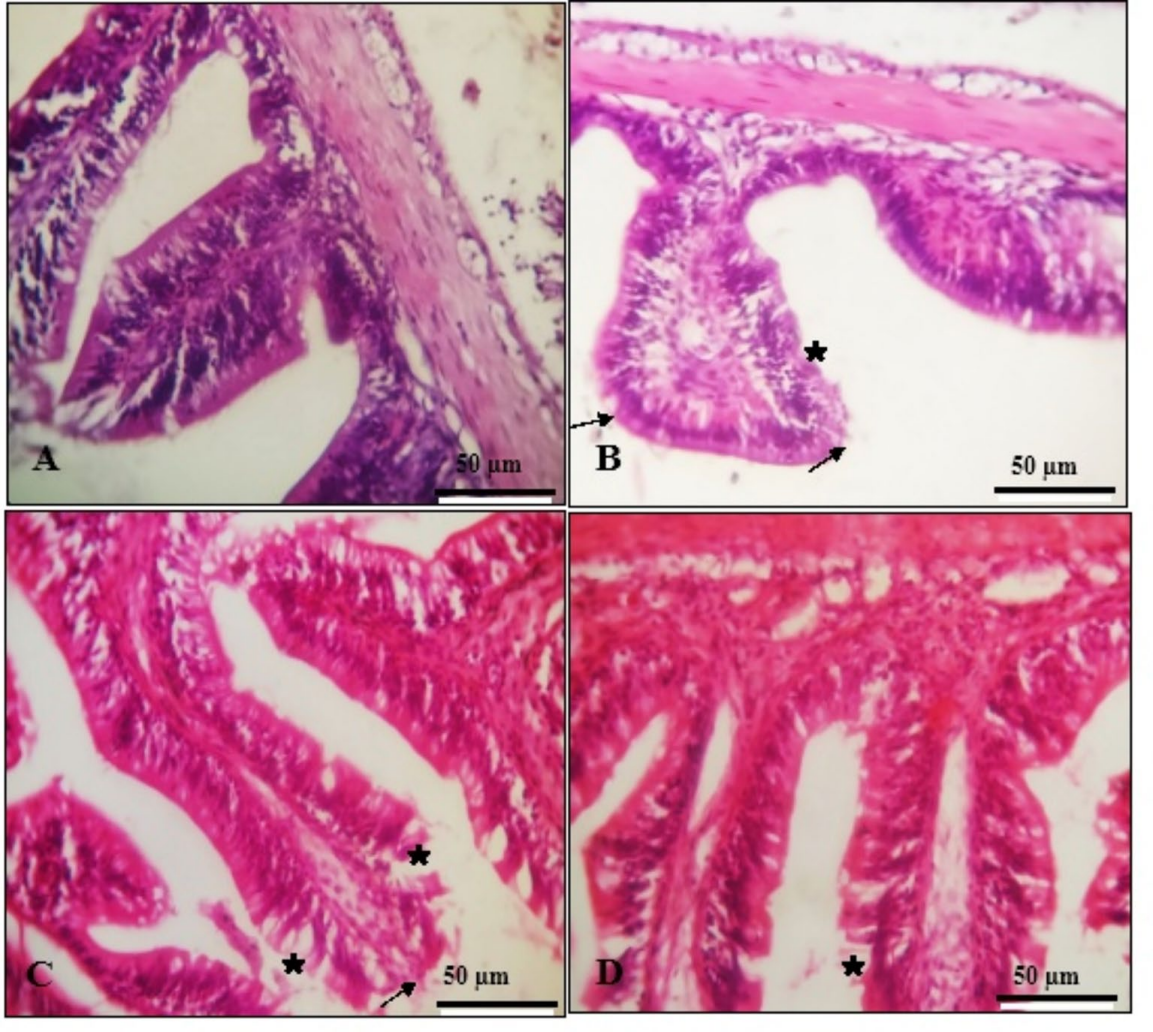
Transverse sections in fish intestine show normal structure in control group (**A**), light deformation in villi cell structure (arrows) with degeneration of their surface epithelial cells (stars) Nano Se group (**B**); vitamin E group (**C**), their normal structure with marked improvement with longing the villi length Nano Se + VE group (**D**) (H&E staining, ×400 magnification, scale bar 50 μm)

Histological analysis of the gut in control fish revealed typical gut anatomy (Fig. 2A) with well-formed villi and goblet cells. Fish fed diets containing VE and SeNPs displayed slight structural distortion in their villi, with degeneration of surface epithelial cells (Fig. 2B and C). In contrast, the SeNPs + VE group showed normal structure with noticeable improvements, including longer villi (Fig. 2D).

#### Hepatopancreas

**Fig. 3:**
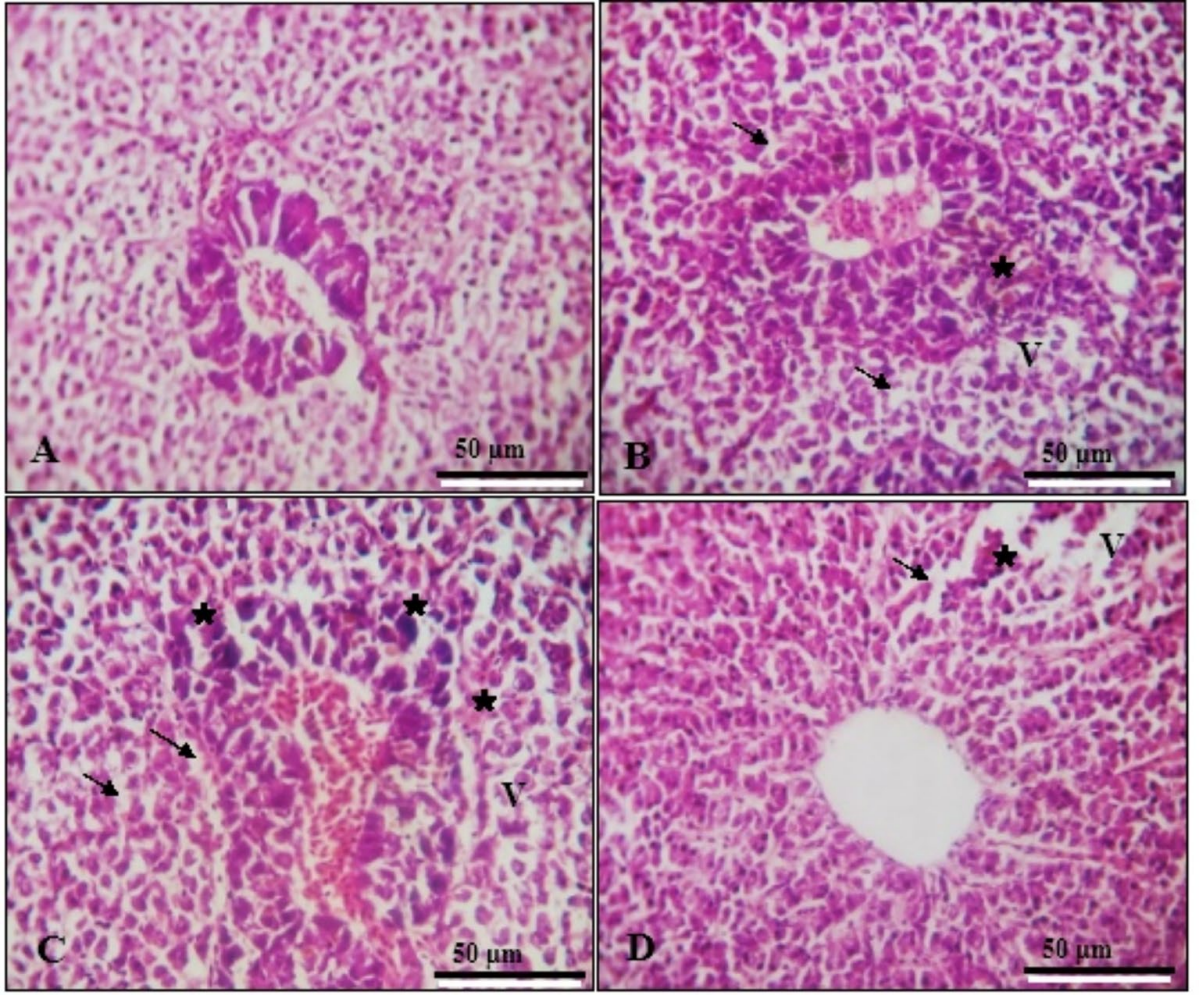
Transverse Sections in fish hepatopancreas show normal structure of its cells hepatic cords, pancreatic acini and normal vascular tissue Control group (**A**), normal morphology of portal veins and hepatic sinusoids were also observed with some vacuolated hepatocytes (V), degeneration of hepatocytes (arrows), the pancreatic islands showed mild congestion (stars) Nano Se group (**B**); vitamin E group (**C**) and Nano Se + VE group (**D**). (H&E staining, ×400 magnification, scale bar 50 μm)

Sections from the hepatopancreas of the control group showed normal cellular structure with hepatic cords, pancreatic acini, and vascular tissue (Fig. 3A). In the SeNPs group (Fig. 3B), some vacuolated hepatocytes (V), hepatocyte degeneration, and mild congestion in the pancreatic islands were observed. Similar changes were seen in the (VE) group (Fig. 3C) and the SeNPs + VE group (Fig. 3D), with normal morphology of portal veins and hepatic sinusoids.

### Challenge assays with fungal infection

**Fig. 4:**
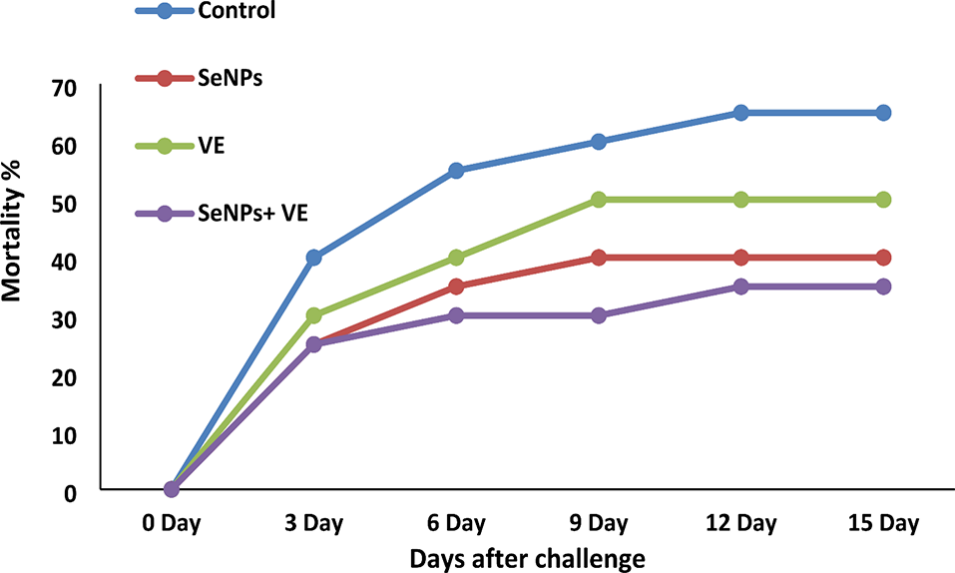
Mortality of Nile tilapia (*Oreochromis niloticus*) after *Aspergillus flavus* infection

The fungal pathogen *A. flavus* was used to induce the fungal infection. Figure 4 shows the cumulative mortality rates of Nile tilapia challenged with *A. flavus* and fed SeNPs, VE, SeNPs + VE, or a control diet. After the *A. flavus* challenge, dietary supplementation with SeNPs, VE, or the SeNPs + VE combination significantly reduced mortality. The control group had the highest mortality rate at 65%. The SeNPs, VE, and SeNPs + VE groups showed mortality rates of 35%, 45%, and 30%, respectively, indicating a decrease in mortality. Notably, the SeNPs + VE group had the lowest mortality rate compared to the control. Fish began to die on the third day following the challenge. Infected fish exhibited increased mucus discharge, scale separation, and bleeding in multiple areas of their external body surface. Postmortem examination revealed an enlarged gall bladder and a pale, swollen liver with white nodules on its surface.

## Discussion

To support the growth and health of farmed fish, it is essential to include micro/nano nutrients in prepared diets in appropriate amounts [53–56]. Our findings demonstrated that SeNPs and/or VE significantly improved the final body weight (FBW), weight gain (WG), and specific growth rate (SGR) in Nile tilapia. These results align with previous studies investigating the effects of selenium supplementation in various fish species [57–59]. Specifically, selenium-fed Nile tilapia have shown notable improvements in growth performance [14]. This study supports the idea that lower concentrations of microminerals, when provided in nanoform, can achieve growth outcomes comparable to or better than those achieved with inorganic and organic forms of these nutrients [60].

Most researchers agree that fish can absorb SeNPs more efficiently than other selenium sources, which may also produce stronger biological effects [61, 62]. In this study, feeding Nile tilapia with SeNPs combined with dietary VE improved their growth and feed efficiency. Similarly, in grass carp, beluga, and black sea bream, VE significantly enhanced (WG) by reducing the (FCR) [63, 64]. Diets low in VE can lead to lipid peroxidation, altering the flavor and reducing the palatability of the feed [65]. Additionally, combining VE with selenium improved growth and feed utilization in largemouth bass, hybrid striped bass, and yellowtail kingfish [66].

Moisture, crude protein, and crude lipid content in target organs are commonly used to indicate the effectiveness of feed additives and the nutritional status of fish [67]. Unlike the findings of Naderi et al. [68], where muscle dry matter, moisture, crude lipid, and crude ash were unaffected by dietary SeNPs, VE, or their combination, the current study observed a slight increase in the whole-body composition of Nile tilapia—including moisture, protein, lipid, and ash content—when supplemented with SeNPs, VE, or their interaction compared to the control group. Similarly, Le et al. [69] reported that dietary selenium, VE, and their combination had no significant impact on moisture, protein, fat, and ash content in yellowtail kingfish (*Seriola lalandi*) fillets, aligning with our results. However, Sang et al. [70] found that liver lipid and flesh protein levels in Snubnose Dart (*Trachinotus blochii*) fed organic selenium diets were higher than those of the control group. Additionally, Li et al. [71] demonstrated that largemouth bass fed diets with varying concentrations of VE had significantly higher crude protein content in their bodies and muscles compared to those fed a basal diet. These findings underscore the variable effects of dietary selenium and VE supplementation across fish species, potentially influenced by differences in species-specific nutrient requirements and feed formulations.

Dawood et al. [42] reported that the blood biochemical parameters observed in this study are consistent with those typically found in Nile tilapia. According to Hoseinifar et al. [48], serum protein, albumin, and globulin play crucial roles in the immune response. Blood total protein levels and serum proteins are indicators of an improved fish immune system [72]. In this study, the elevated blood total protein levels suggest that VE and/or SeNPs have immunomodulatory effects on Nile tilapia. This indicates that dietary SeNPs and/or VE can boost the health of farmed tilapia. Furthermore, the study found that all experimental groups, except the VE group, showed higher serum albumin levels. Measuring globulin levels provides valuable diagnostic insights into fish health [73]. All experimental groups demonstrated significantly higher serum globulin concentrations compared to the control group.

The liver enzymes ALT, AST, and ALP are key indicators of liver health, with elevated levels typically associated with liver disease [9]. This study found that all treatment groups showed the lowest serum activities of AST, ALT, and ALP, indicating minimal damage to normal liver function. Blood glucose, produced as a secondary response to increased energy demands during stress, serves as a marker of stress response [74]. In this study, fish fed with VE or SeNPs had significantly lower glucose levels compared to the control group. This suggests that incorporating SeNPs and/or VE into the diet of *O. niloticus* offers additional stress-reducing benefits. Similarly, serum creatinine, a marker of renal health that increases during nephrotoxicity, was lower in fish fed SeNPs and/or VE compared to the control group [9, 75].

Fish lysozyme, found in lymphoid tissue, mucus, plasma, and other body fluids, exhibits lytic activity against both Gram^+^ and Gram^−^ bacteria [76]. In this study, serum lysozyme activity in Nile tilapia was higher in the treatment groups compared to the control group. This contrasts with findings in fancy carp, which showed reduced lysozyme activity [77]. Belo et al. [78] reported that vitamin E protects fish from stress-induced immunosuppression. Consistent with Naderi et al. [68], our results showed that fish fed diets supplemented with VE (VE and combination groups) had the highest lysozyme activity levels. Both selenium and VE are essential for protecting fish against oxidative stress [57, 66].

Kandeil et al. [79] identified SOD, CAT, and MDA as key indicators for assessing antioxidant function in animals. Superoxide dismutase (SOD) and CAT play critical roles in scavenging free radicals (ROS) and prevent lipid peroxidation damage. Malondialdehyde (MDA), a harmful compound generated during lipid peroxide breakdown, reflects the extent of cell damage and lipid peroxidation [80]. In this study, fish fed with either VE or SeNPs showed higher SOD and CAT activities, suggesting reduced cellular damage, and lower MDA levels in all treatment groups except for SeNPs. Similarly, supplementing diets with SeNPs or VE has been shown to enhance antioxidant responses in various fish species [81].

According to Kumar et al. [82], histopathology provides a rapid method to assess the effects of irritants on various organs. Exposure to chemical pollutants is known to cause a range of lesions in organs such as the liver and gills [83]. Figures 1 and 3 illustrate the histology of the gills and hepatopancreas in *O. niloticus* after exposure to SeNPs and VE. The results showed generally normal liver and gill architecture, with some observable abnormalities. These included vacuolated hepatocytes, mild degeneration of hepatocytes, congestion in the pancreatic islands, and laceration of some gill arches. Additionally, hyperplasia and fusion of the primary and secondary gill lamellae were noted in the SeNPs and VE-treated groups.

These findings align with Kumar et al. [84], who observed similar abnormalities in the liver and gills of iridescent shark (*Pangasianodon hypophthalmus*) exposed to selenium and SeNPs. Reported changes included large vacuoles, cloudy swelling, focal necrosis, interstitial edema, hemorrhage, necrosis, pyknotic nuclei, and hepatocyte hypertrophy. Additionally, alterations in the gills included fusion of secondary lamellae, curling of secondary lamellae, and thickening of the primary lamellae epithelium. Ozardali et al. [85] also reported liver damage resulting from selenium exposure, while Wang et al. [86] documented comparable effects in animals exposed to selenium and SeNPs.

In this study, the villi surface epithelial cells showed mild degeneration when exposed to the SeNPs and VE groups. However, *O. niloticus* subjected to SeNPs and VE showed significant improvement in their normal structure, with the villi becoming longer (Fig. 2). Significant improvements (*P* *< 0.05*) were observed in villus height, villus width, muscular thickness, and goblet cell count in fish fed the SeNPs + VE diet. These improvements are attributed to the interaction between SeNPs and VE (Table 6). These results are consistent with those of Dawood et al. [42], who observed increased intestinal villus heights and goblet cells in fish fed diets supplemented with SeNPs and/or VE. These findings suggest an enhanced absorptive surface area, which improves nutrient absorption, boosts resistance to invasive intestinal pathogens, and may act as a growth promoter. Therefore, the increased protein content in intestinal cells, leading to better nutrient consumption, may explain the improved feed efficiency in tilapia fed SeNPs and/or VE. The hepatic cells in this study also demonstrate the positive impact of SeNPs and/or VE on the morphometry of the epithelial mucosa, which may contribute to improved nutrient retention and absorption.

**Table 6:** Intestinal biometric indices of Nile tilapia (*O. Niloticus*) for four experimental feeds

Parameters	Control	SeNPs	VE	SeNPs + VE
Villus height (µm)	291.23^a^ ± 0.47	243.00^b^ ± 0.53	244.66^b^ ± 0.88	291.47^a^ ± 0.73
Villus width (µm)	88.56^a^ ± 0.23	55.33^b^ ± 0.88	54.83^b^ ± 0.16	88.84^a^ ± 0.20
Muscular thickness (µm)	35.15^b^ ± 0.42	30.86^c^ ± 0.57	30.91^c^ ± 0.23	40.74^a^ ± 0.33
Goblet cells count	33.00^a^ ± 0.57	25.66^b^ ± 0.33	25.00^b^ ± 0.57	34.00^a^ ± 0.58

Means having different letters in the same row are significantly different at *P* < 0.05

Selenium is a micronutrient that works with other nutrients as a co-enzyme to produce and activate digestive enzymes [87]. Previous studies have shown that dietary selenium increases the quantity and activity of intestinal bacteria and digestive proteases, improving protein digestibility and utilization [88]. After exposure to *A. flavus*, *O. niloticus* exhibited clinical symptoms, postmortem changes, and histological indicators consistent with a recent study [38]. This study demonstrated the protective effects of SeNPs and VE in Nile tilapia culture, offering an alternative to antifungals for preventing *A. flavus* infection. Tilapia fed with SeNPs and VE (T3) showed a significantly lower cumulative mortality rate during the challenge test.

## Conclusion

Fish immunity and health are influenced by vitamin E and SeNPs in various ways. The *O. niloticus* showed increased resistance to *A. flavus* following treatment with SeNPs and VE. This study highlights how nanotechnology can be applied to improve Nile tilapia farming, potentially contributing to addressing the global food crisis. Fish farming enterprises may consider producing nano-feed additives with optimal levels of VE and selenium to enhance fish health.

## Data Availability

All data regarding this study are presented in the paper.
